# Usefulness of virtual enteroscopy for the detection of small polypoid lesion in the small bowel, a case report

**DOI:** 10.1016/j.ijscr.2020.01.009

**Published:** 2020-01-23

**Authors:** Ryoma Haneda, Shinsuke Sato, Kazuya Ohno, Toshiyuki Yoshikawa, Masakazu Takagi

**Affiliations:** Shizuoka General Hospital, Shizuoka, Japan

**Keywords:** VE, virtual enteroscopy, CT, computed tomography, VCE, video capsule endoscopy, BE, balloon endoscopy, Virtual enteroscopy, Video capsule endoscopy, Polypoid lesion, Pyogenic granuloma, Case report

## Abstract

**Introduction:**

Virtual enteroscopy (VE) has been developed to explore the entire small bowel. We have previously reported that VE can reveal elevated lesions measuring >10 mm in diameter. However, data on the existence of smaller polypoid lesions is scarce. This study aimed to report a case of pyogenic granuloma in the ileum detected by VE.

**Presentation of case:**

A 55-year-old woman presented to our hospital with iron deficiency anemia. Esophagogastroduodenoscopy, colonoscopy, and abdominal contrast-enhanced computed tomography did not indicate any bleeding sources. Video capsule endoscopy revealed a small polypoid lesion in the small bowel. VE was subsequently performed and a polypoid lesion was detected at 119 cm from the ileocecal valve. Its size was estimated to be 6 mm. Based on VE findings, laparoscopic-assisted surgery for the small bowel tumor was performed. During surgery, the polypoid lesion, at 120 cm from the end of the ileum, was barely palpable. The resected specimen showed a 5.5 × 5.0 mm polypoid lesion. Microscopically, the polypoid lesion was diagnosed as pyogenic granuloma.

**Discussion:**

We detected a 5.5 × 5.0 mm polypoid lesion in the small bowel, and this is the minimum size of the lesion visualized on VE. This imaging technique provides surgeons with data on the location, number, and size of polypoid lesions.

**Conclusion:**

VE is a new useful tool for the preoperative collection of data on small polypoid lesions in the small bowel.

## Highlights

• A small polypoid lesion in the small bowel was detected by virtual enteroscopy (VE).
• VE imaging technique provides surgeons with data on the location, number, and size of polypoid lesions.
• Based on VE findings, a 5.5 × 5.0 mm pyrogenic granuloma was resected successfully by laparoscopic-assisted surgery.

## 1 Introduction

Small polypoid lesions in the small bowel are difficult to detect and locate. Although small bowel barium follow-through, double-contrast enteroclysis, and computed tomography (CT) are conventionally performed, visualization of small polypoid lesions in the small bowel remains inadequate. Recently, video capsule endoscopy (VCE) and balloon endoscopy (BE) have also been developed to detect small bowel lesions [1,2]. However, these methods suffer from some disadvantages, such as inaccuracy in the locating the lesions, invasiveness of BE, and accidental capsule retardation with VCE.

Virtual enteroscopy (VE) has been developed in our hospital to explore the entire small bowel by inflating it with air and depicting the wall using CT colonographic system. Here, we report a case of pyogenic granuloma in the ileum detected by VE. The work has been reported in line with the SCARE criteria [3].

## 2 Presentation of case

A 55-year-old woman presented to our hospital with shortness of breath. She was diagnosed with anemia 2 years ago, which improved upon intake of iron supplements but recurred shortly after stopping supplementation. She had no significant past medical history and physical findings.

**Fig. 1:**
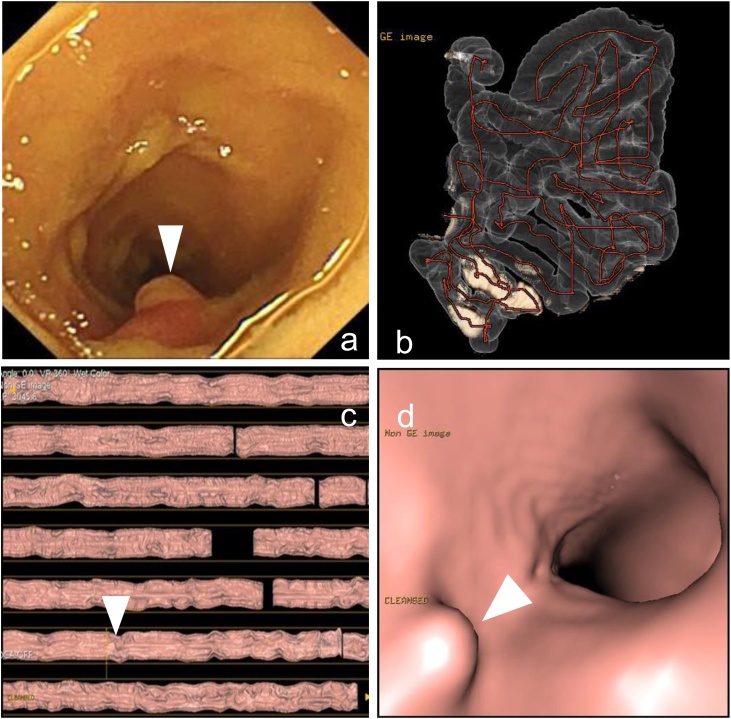
Video capsule endoscopy (VCE) and virtual enteroscopy (VE). VCE (a) revealed a small, red polypoid lesion in the ileum (arrowhead). Three-dimensional overview (b), dissection view (c), and virtual endoscopic view (d) showed a 6-mm polypoid lesion (arrow heads) in the ileum at 119 cm from the ileocecal valve.

Laboratory data indicated iron deficiency anemia. Esophagogastroduodenoscopy, colonoscopy, and abdominal contrast-enhanced CT did not show any bleeding sources. VCE revealed a small polypoid lesion in the small bowel (Fig. 1a).

VE was subsequently performed to depict and locate the small bowel lesion. An 18Fr nasojejunal tube with double balloon (Faicon hydrophilic ileus tube set with double balloon; Fuji Systems, Tokyo, Japan) was inserted beyond the pylorus. The anterior balloon was inflated at the duodenal bulbus, whereas the posterior balloon was inflated at the gastric antrum. Air was slowly injected at a rate of 200 mL/min. A 64-channel multi-detector row CT (LightSpeed VCT; GE Healthcare Japan, Tokyo, Japan) was employed. Image processing was performed using a commercially available CT colonographic system (Advantage Workstation; GE Healthcare, Milwaukee, WI, USA). The small bowel, which was shown in three-dimensional overview (Fig. 1b), dissection view (Fig. 1c), and virtual endoscopic view (Fig. 1d), was 469 cm in length from the pylorus and 445 cm from ligament of Treitz. A polypoid lesion was detected in the ileum at 119 cm from the ileocecal valve. Its size was estimated to be 6 mm in the virtual endoscopic view. Considering the VE findings, retrograde single-balloon endoscopy for polypectomy was performed. However, the scope could not reach the polypoid lesion for polypectomy.

**Fig. 2:**
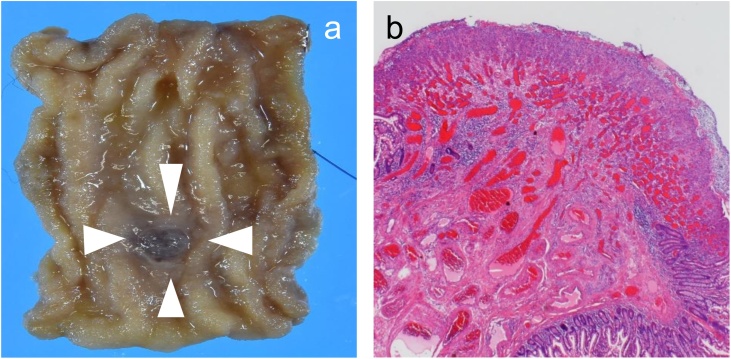
Histopathological images. Loupe images of surgical specimen (a) and hematoxylin and eosin-stained section (b).　Growth of capillaries was observed in the mucosa. In the submucosal layer, arteriovenous growth was also detected. Histopathological diagnosis was pyogenic granuloma.

Subsequently, laparoscopic surgery was performed. The small bowel was 450 cm in length from the ligament of Treitz, when measured by forceps. The ileum was exteriorized at 120 cm from the ileocecal valve through an umbilical incision based on the VE findings. The polypoid lesion was palpable at almost the same location. Partial resection and end-to-end anastomosis were performed. The resected specimen showed a 5.5 × 5.0 mm polypoid lesion. Histopathological diagnosis was of pyogenic granuloma (Fig. 2a and b). The patient had a good postoperative course and was discharged 6 days after surgery. No significant postoperative complication and anemia was noted.

## 3 Discussion

The VE protocol was established in our hospital, and 177 examinations have been performed by 2018. In the present study, the procedures were completed in all cases without incidence of any serious complications. The invasiveness of VE is equivalent to that of double-contrast small bowel series, because both examinations require intubation and injection of contrast material and air. The success rate of total enteroscopy varies among investigations, as observed by Gross and Stark [4] and Ohmiya et al. [5] who reported a success rate of 20% in 45 patients and 61% in 204 patients, respectively, with antegrade and retrograde approaches. VE can trace the entire small bowel in approximately 70% of cases in a single procedure. With respect to success rates, VE could be a superior modality for the observation of the entire small bowel.

Several elevated lesions (including malignant lymphoma, gastrointestinal stromal tumor, small bowel carcinoma, benign polyps, Peutz-Jeghers syndrome, inverted Meckel’s diverticulum, and lipoma) have been detected by VE in our hospital [6]. With the help of virtual colonoscopy program, we can measure the size of lesions using VE. We previously reported that VE could reveal elevated lesions measuring >10 mm in diameter [7], but failed to detect polypoid lesions measuring <5 mm. In the present case, the polyp had a size of 5.5 × 5.0 mm and was clearly detected by VE. This is the minimum lesion size that was visualized by VE.

Pyogenic granulomas can cause gastrointestinal bleeding, due to their hypervascular nature. It is reported that selective angiography of the celiac artery clearly revealed a hypervascular lesion and transarterial embolization of the nutritional artery of the lesion was conducted for pyogenic granuloma in the stomach successfully [8]. Nutritional artery could have been identified, if contrast enhanced abdominal CT had been done for VE.

VE is the only examination that can locate lesions objectively. With balloon enteroscopy, the information is subjective. In VCE, the capsule goes backward and forward in the intestinal lumen, hence, the data on location, size, and number of lesions are imprecise. The site of the lesion can be precisely located with VE, because it can be described with respect to the distance from the ligament of Treitz or ileocecal valve and mapped in dissection view. This modality is very useful for surgeons, as it aids in locating small lesions during surgery, which is an advantage over any other imaging technique, including VCE and BE.

## 4 Conclusion

In this case, a small polypoid lesion with a size of 5.5 × 5.0 mm was detected in the small bowel by VE. VE imaging technique could become a new way for surgeons to plan for minimally invasive surgery, which contribute to the preservation of quality of life.

## Sources of funding

This research did not receive any specific grant from any funding agencies in the public, commercial, or not-for-profit sectors.

## Ethical approval

The authors declare that we obtained permission from ethics committee in our institution.

## Consent

Written informed consent for publication of this case report with accompanying images was obtained from the patient.

## Author contribution

Ryoma Haneda: Conceptualization; Data curation; Investigation; Visualization; Writing - original draft.

Shinsuke Sato: Conceptualization; Methodology; Project administration; Writing - review & editing.

Kazuya Ohno: Conceptualization; Data curation.

Toshiyuki Yoshikawa: Conceptualization; Data curation; Methodology; Writing - review & editing.

Masakazu Takagi: Supervision.

## Registration of research studies

We enter the name of registry, and the unique identifying number (UIN) of our study is researchregistry5164.

## Guarantor

Shinsuke Sato.

## Provenance and peer review

Editorially reviewed, not externally peer-reviewed.

## Declaration of Competing Interest

All authors declare that they have no competing interests.
